# Retraction: Fourier Transform Infrared Spectroscopy vibrational bands study of *Spinacia oleracea* and *Trigonella corniculata* under biochar amendment in naturally contaminated soil

**DOI:** 10.1371/journal.pone.0272183

**Published:** 2022-08-03

**Authors:** The PLOS ONE Editors

The *PLOS ONE* Editors retract this article [1] because it was identified as one of a series of submissions for which we have concerns about authorship, competing interests, and peer review. We regret that the issues were not addressed prior to the article’s publication.

UY, AAR, SD, MAA, RD, SF, JH, TH, MB, TZ, AES and BRG did not agree with the retraction. NA and AB either did not respond directly or could not be reached.
